# Supplementary material for: Deep multimodal representations and classification of first-episode psychosis via live face processing
Source: Front Psychiatry. 2025 Feb 26;16:1518762. doi: 10.3389/fpsyt.2025.1518762 (PMC11934110; doi:10.3389/fpsyt.2025.1518762)
Supplement: Supplementary file 1 [file SupplementaryFile1.pdf]

A Path Signatures

The path signature is a structured summary of a path in a multidimensional space, characterizing its properties by capturing all iterated integrals of the path components up to a specified truncation order. Following the formalism presented by Chevyrev and Kormilitzin (Chevyrev and Kormilitzin, 2016), given a piecewise differentiable path $X : [a, b] \to \mathbb{R}^d$, we define the signature of X, denoted $S(X)_{a,b}$, is an infinite sequence where each term is derived from the iterated line integrals of X. Specifically, the k-th level signature, $S^{(k)}(X)_{a,b}$, is given by:

$$S^{(k)}(X)_{a,b} = \int_{a<t_1<\cdots<t_k<b} dX_{t_1} \otimes \cdots \otimes dX_{t_k} \tag{1}$$

This sequence starts from $k = 1$ and continues indefinitely, capturing increasingly complex interactions among the path components over its domain. To compute this in practice, especially for paths represented by discrete data points $X_{t_0}, X_{t_1}, \ldots, X_{t_n}$ with $t_0 = a$ and $t_n = b$, the iterated integrals are approximated by summing over all ordered combinations of the sampled points:

$$S^{(k)}(X)_{a,b} \approx \sum_{i_1<i_2<\ldots<i_k} (X_{t_{i_1+1}} - X_{t_{i_1}}) \otimes \cdots \otimes (X_{t_{i_k+1}} - X_{t_{i_k}}) \tag{2}$$

In order to manage computational costs, the signature is typically truncated at a finite level N, providing a compact yet informative representation:

$$S^N(X)_{a,b} = \left(1, S^{(1)}(X)_{a,b}, \ldots, S^{(N)}(X)_{a,b}\right) \tag{3}$$

Each term of $S^N(X)_{a,b}$ is a tensor product of differences between successive path points, efficiently encapsulating the path's essential geometric and dynamic features up to the truncation level.

**Feature**	**EEG Band**	**Unimodal**	**Multimodal**	
			EEG + fNIRS	EEG + FaceAU
Curvature	alpha	0.586	0.633	0.695
	delta	0.587	0.726	0.678
	theta	0.632	**0.753**	0.729
Path Signature	alpha	0.514	0.557	0.583
	delta	0.509	0.764	0.772
	theta	0.596	**0.780**	**0.786**

Table 1: Leave-one-out classification accuracies based on curvature and path-signatures from 3 dimensional t-PHATE embeddings obtained from unimodal and multimodal latent trajectories generated by neural-PRISM with input from various EEG bands.

B Multimodal Translation

Here we present the results on modality translation which is an additional outcome of our Neural-PRISM framework. We randomly hold out 10% of samples for testing while the rest of the samples were used for training. The average root mean square errors and correlations are shon in Figure 1 (a). Some example translated fNIRS signals are depicted in Figure 1 (b). Figure 1 (c) shows the average Spearman correlation coefficients of the translated signals from respective EEG bands. The averaged root mean square errors in translated FaceAUs from the corresponding EEG data are depicted in Figure 2 (a). Figure 2 (b) shows a sample reconstructed face (as x,y, z locations) from predicted FaceAUs. This face reconstruction is obtained using the learned linear regressor from ground truth training data. Note that the translation of different modalities is a side product of Neural PRISM framework. Further study of the effect of different action units from EEG or fNIRS data is an interesting problem, which we plan to explore in future.

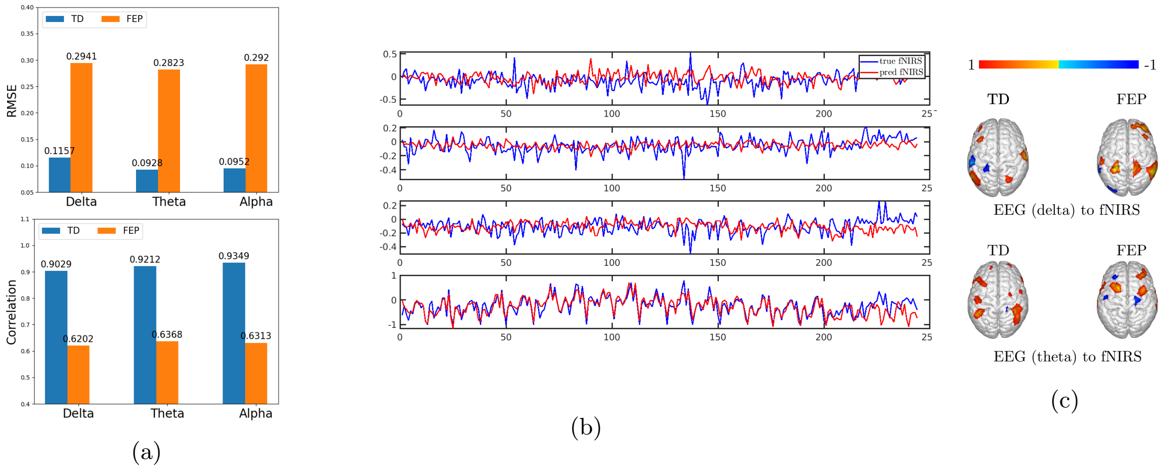

Figure 1: (a) Correlations and root mean square errors (RMSE) for translation from EEG to fNIRS, corresponding to different EEG bands. (b) Example reconstructed fNIRS (functional near-infrared spectroscopy) samples and (c) Average correlations for reconstructed fNIRS samples corresponding to TD (typically developed) and FEP (first episode psychosis) subjects.

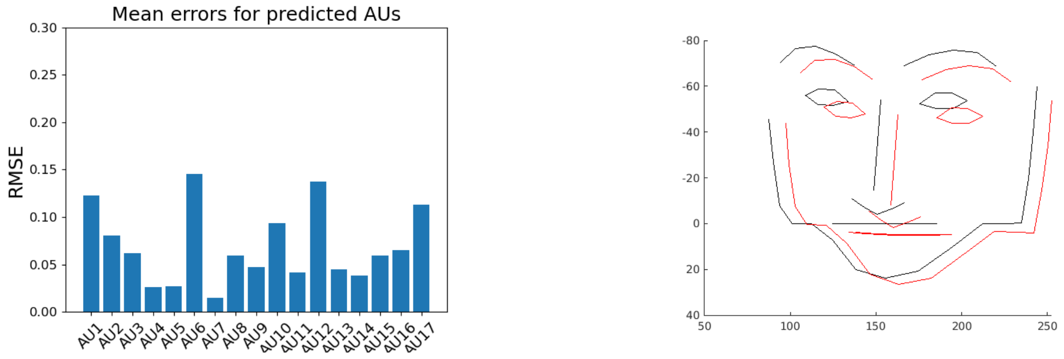

(a) (b)

Figure 2: (a) RMSE (root mean square error) values for the different predicted action units. (b) Sample reconstruction from predicted FaceAUs (fiacial action units). Red color represents predicted face while blue color represents the actual facial configuration (ground truth). The reconstruction of facial configuration is a validation of FaceAU predictions.
